# Enzymatic synthesis of kraft lignin-acrylate copolymers using an alkaline tolerant laccase

**DOI:** 10.1007/s00253-022-11916-z

**Published:** 2022-04-22

**Authors:** Maryam Arefmanesh, Thu V. Vuong, Saeid Nikafshar, Henrik Wallmo, Mojgan Nejad, Emma R. Master

**Affiliations:** 1 Department of Chemical Engineering and Applied Chemistry, University of Toronto, 200 College Street, Toronto, ON M5S 3E5 Canada; 2 Department of Forestry, Michigan State University, 480 Wilson Road, East Lansing, MI 48824 USA; 3 Valmet AB, Regnbågsgatan 6, PO Box 8734, 402 75 Gothenburg, Sweden; 4 Department of Chemical Engineering and Material Science, Michigan State University, 428 S Shaw Lane, East Lansing, MI 48824 USA; 5 Department of Bioproducts and Biosystems, Aalto University, Kemistintie 1, 00076 Aalto Espoo, Finland

**Keywords:** Kraft lignin, Laccase, Grafting, Copolymerization, Polyol, Polyurethane

## Abstract

**Abstract:**

Softwood kraft lignin is a major bioresource relevant to the production of sustainable bio-based products. Continued challenges to lignin valorization, however, include poor solubility in organic solvents and in aqueous solutions at neutral pH. Herein, an alkaline tolerant laccase was used to graft acrylate functionalities onto softwood kraft lignin, which is expected to enhance the reactivity of lignin with isocyanate when producing bio-based polyurethanes. Proton nuclear magnetic resonance, Fourier-transform infrared spectroscopy, and high-performance liquid chromatography were used to confirm successful grafting of the acrylate monomer onto lignin and verify the importance of including tert-butyl hydroperoxide as an initiator in the grafting reaction. Laccase-mediated grafting of softwood kraft lignin under alkaline conditions produced lignin products with approximately 30% higher hydroxyl value and higher reactivity toward isocyanate. The reported enzymatic and aqueous process presents an opportunity for the sustainable valorization of softwood kraft lignin.

**Key points:**

• *Softwood kraft lignin displayed high phenolic hydroxyl content, polydispersity index and average molecular weight*

• *Grafting hydroxyethyl acrylate (HEA) monomer onto kraft lignin by laccase was successful at 60 °C and alkaline conditions*

• *Lignin-HEA grafted copolymer showed an increase in total OH value and an increase in average molecular weight*

**Supplementary information:**

The online version contains supplementary material available at 10.1007/s00253-022-11916-z.

## Introduction

Lignin is the most abundant naturally occurring aromatic polymer and represents 15 to 30 wt% of the available carbon in lignocellulosic biomass (Laurichesse and Avérous 2014; Ragauskas et al. 2014; Ralph et al. 2019; Yoo and Ragauskas 2021). Lignin is widely recovered through chemical pulping with an annual production of approximately 70 Mt/y in 2018 and projected increase to 225 Mt/y by 2030 (Bajwa et al. 2019; Dessbesell et al. 2020); accordingly, its utilization is critical to the development of renewable bio-based materials (Yang et al. 2021; Sun et al. 2020). Kraft lignin is by far the most abundant source of technical lignin and is recovered from black liquor generated during the kraft pulping process (Chakar and Ragauskas 2004; Ragauskas et al. 2014; Crestini et al. 2017). The harsh alkaline conditions required for kraft pulping breakdown lignin structures and condense the resulting fragments (Cui et al. 2014; Crestini et al. 2017). Consequently, major hurdles to the valorization of kraft lignins include high heterogeneity and low solubility in most solvents.

Polyurethanes (PU) represent a large class of polymers with urethane repeating units that are produced by reacting polyols and diisocyanates; the global market for PUs is expected to grow from USD 44 billion in 2018 to USD 59 billion in 2024 (Mordor Intelligence 2019). Growing environmental and economic concerns regarding petroleum-based polyols (and isocyanates) motivate the search for alternatives. For example, bio-based polyols are especially sought after for the production of PU binders used to manufacture wood-based panels, coatings, and foams (Akindoyo et al. 2016). Accordingly, the bio-based polyol market is estimated to reach USD 11 billion by 2025 (IndustryARC 2021) and kraft lignin could represent a major renewable resource to meet this demand.

**Scheme 1:**
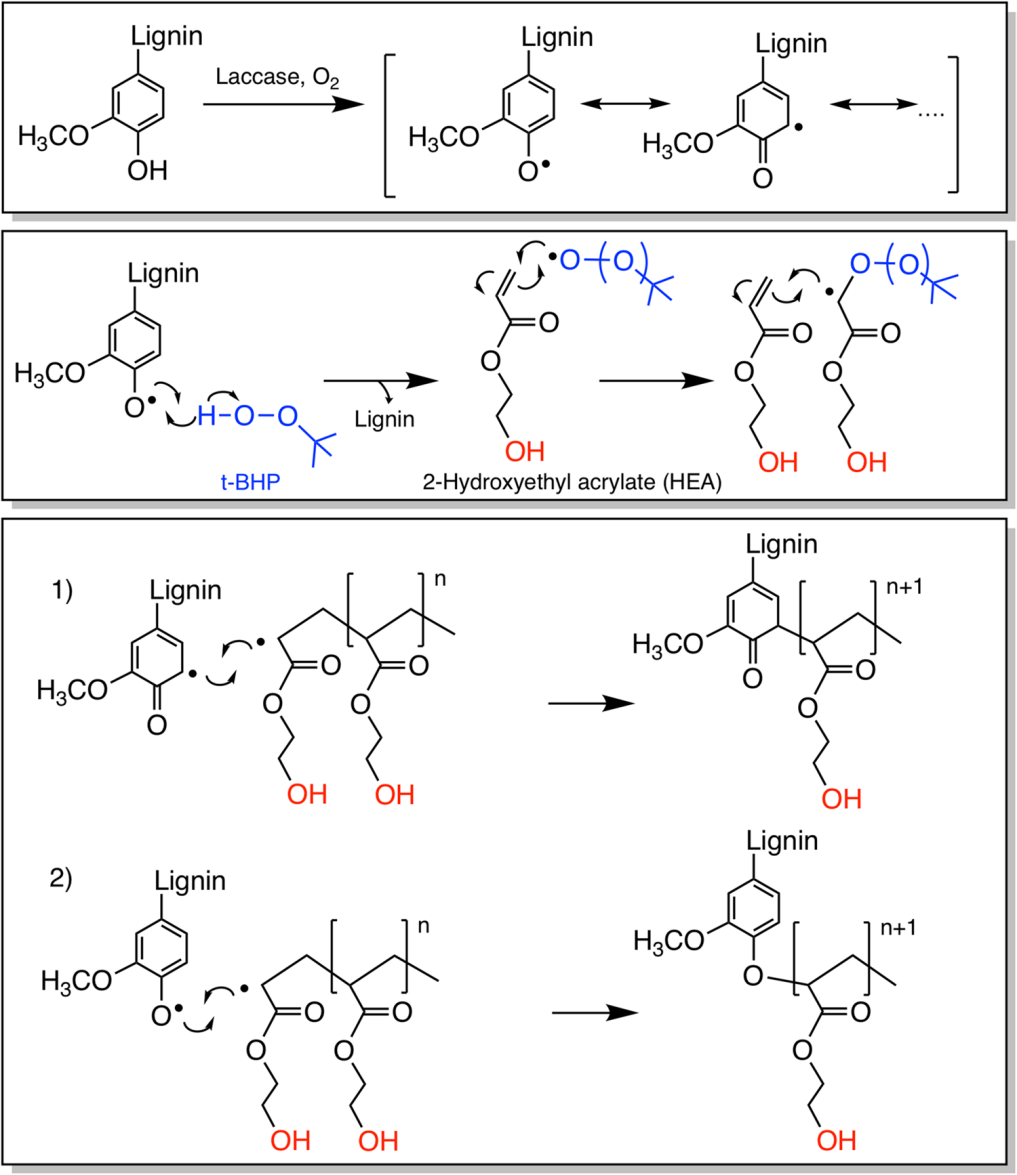
Proposed mechanism of laccase-initiated copolymerization of lignin and acrylate monomer. Phenoxy radicals that are formed by laccase oxidation in the presence of oxygen (top panel) activate the initiator tert-butyl hydroperoxide (t-BHP, in blue), initiating 2-hydroxyethyl acrylate (HEA) polymerization (middle panel, the hydroxyl group of HEA is in red), which is quenched by radicals on lignin, following route 1 and/or 2 (bottom panel), and consequently grafted on lignin phenoxide (Mai et al. 1999)

The inaccessibility of hydroxyl groups and the complexity as well as heterogeneity of lignins, however, limit its broader application (Hatakeyama and Hatakeyama 2010; Chatel and Rogers 2014; Laurichesse and Avérous 2014). The accessibility of aliphatic hydroxyl groups in lignin is especially important in PU applications (Delebecq et al. 2013; Alinejad et al. 2019) and can be achieved through the methylolation (Meister 2002) or hydroxyalkylation (Wu and Glasser 1984) of lignin structures. An alternative approach is to graft aliphatic hydroxyl groups onto lignins (Eraghi Kazzaz et al. 2019; Lin et al. 2014). Several studies were carried out to copolymerize lignin with various vinyl monomers using initiators such as potassium persulfate (Alipoormazandarani and Fatehi 2020) or the sodium thiosulfate/potassium persulfate system (Kong et al. 2015). As these chemical systems require a continuous supply of nitrogen for deoxygenation, chemo-enzymatic initiator systems were developed for laccase-mediated grafting of acrylic acid or acrylamide to lignosulfonate in the presence of the initiator tert-butyl hydroperoxide (t-BHP) (Mai et al. 2000a, 2002; Yu et al. 2017). In these reactions, phenoxy radicals generated by laccase react with t-BHP to create alkoxy/peroxy radicals that initiate the homopolymerization of acrylic acid or acrylamide (Scheme 1). Extension of the acrylate chain is terminated through covalent bond formation with a phenoxy radical (Mai et al. 1999; Yu et al. 2017).

To investigate the feasibility of laccase-mediated grafting of kraft lignin, an alkaline-active laccase was evaluated for its potential to graft 2-hydroxyethyl acrylate (HEA) to softwood kraft lignin, thereby increasing the suitability of kraft lignin as a polyol in PU formulations. We demonstrate the enzymatic preparation of lignin-HEA copolymers (LHC) in an alkaline condition and report reaction conditions that increase the total hydroxyl content of softwood lignin by > 30%, which in turn increased reactivity with isocyanate.

## Materials and methods

### Materials

Softwood kraft lignin from the LignoBoost process was oven dried at 80 °C for 3 h and then suspended in Milli-Q water before adding sufficient 1 N NaOH to solubilize the lignin at 10% (w/v). The resulting solution was left overnight at room temperature; the solubilized lignin was then dialyzed against Milli-Q water using a 3.5-kDa-cut-off dialysis tubing (Thermo Fisher Scientific, Waltham, MA, USA) to remove low molecular weight phenolic compounds or extractives. HEA (98.5%, cat. no. UN2922) was kindly supplied by BASF (Florham Park, NJ, USA), whereas t-BHP (70%, cat. no. AC180340050), phenyl isocyanate, and dimethyl formamide (DMF) were purchased from Thermo Fisher Scientific (Fair Lawn, NJ, USA). The HEA homopolymer (20% solution in water) was purchased from Scientific Polymer Products Inc. (Ontario, NY, USA).

The commercial laccase Novozym®51003, which was previously shown to directly modify lignin in wood (Jeremic et al. 2014), was obtained from Novozymes (Franklinton, NC, USA). The specific activity of the enzyme was measured by assaying 5 μg/mL laccase with 10 mM 2,6-dimethoxyphenol (DMP, also known as syringol) in 50 mM Tris-buffer pH 8 and pH 11 at 60 °C for 10 min; the formation of coerulignone was measured using ε_469_ = 53,200 M^−1^ cm^−1^ (Breslmayr et al. 2018). Enzyme unit (U) is defined as the amount of enzyme, which at 60 °C and pH 11 oxidizes 1 μmol DMP per minute. Enzymatic stability in grafting reagents was tested by pre-incubating laccase with 0.7% (w/v) HEA and 0.7% (w/v) t-BHP in 50 mM Tris-buffer pH 8 and pH 11 at 60 °C for 4 h, before being assayed on 10 mM DMP.

### Lignin content 

​​The ash content of dried lignin samples was measured according to TAPPI T211om-93. The percent ash content of the isolated-lignin sample (six replicates) was measured after calcination of the sample in a Sybron Thermolyne Furnatrol muffle furnace at 525 °C for 4 h. The carbohydrate contents (xylan and glucan contents) were analyzed following the protocol NREL/TP-510–42618 of the US National Renewable Energy Laboratory (Sluiter et al. 2011).

### Grafting an acrylate monomer to kraft lignin

The copolymerization reaction was carried out at pH 11 and 7% (w/v) solubilized lignin, 0.7% (w/v) t-BHP, 0.7% (w/v) HEA, and 1 U laccase. The lignin and laccase reagents were incubated alone for 4 h at 15 rpm at 60 °C in a rotating incubator (Mini Hybridization Oven MK II, Thermo Hybaid, Franklin, MA, USA) to permit enzymatic oxidation and radical formation. The t-BHP and HEA reagents were then added such that the reaction volume (5 mL) filled the 5-mL reaction tubes to avoid air from the head space (Ligon et al. 2014); and each reaction continued for another 12 h at 60 °C. The same reaction mixture but without one to three reaction components served as controls. To investigate the influence of temperature and monomer concentration on grafting, the copolymerization reaction was conducted at different temperatures (25 °C, 40 °C, and 60 °C) and different concentrations of HEA (0.7% and 7%, v/v).

After incubation, 25 μL of the reaction mixture was sampled and diluted 10 times with Milli-Q water before being vacuum filtered using 0.2-μm membranes. The filtrate was analyzed by high-performance liquid chromatography (HPLC–UV/RI) to investigate the monomer consumption during the grafting reaction. The remaining reaction samples were transferred into 3.5-kDa cut-off dialysis tubing (Thermo Fisher Scientific, Waltham, MA, USA) to remove the unreacted monomer from the reaction mixture. Milli-Q water for dialysis was replaced 3 times after every 2 h at room temperature. The samples were transferred to 15-mL centrifugal tubes and freeze dried for 24 h using a Flexi-Dry MP freeze-dryer (FTS Systems Inc., Stone Ridge, NY, USA). Fourier transform infrared spectroscopy-attenuated total reflectance (FTIR-ATR) and nuclear magnetic resonance spectroscopy (NMR) analyses were performed on the dried solid fraction.

### Isocyanate reactivity

The reactivity of hydroxyl groups in lignin before and after the grafting reaction was measured with phenyl isocyanate. First, dried lignin and phenyl isocyanate (1 to 1, w/w) were dissolved in 10 g dried DMF, and reacted at 50 °C for 1 h under nitrogen atmosphere. Reaction products were then analyzed using FTIR-ATR to identify changes in isocyanate and urethane absorbance bands.

### HPLC analysis

HPLC was conducted using an UltiMate-3000 system (Dionex, Sunnyvale, CA, USA) equipped with an Aminex HPX-87H column (300 mm × 7.8 mm, catalog no. 125–0140). Each sample (20 µL) was injected onto the column, and 5 mM H_2_SO_4_ was used as an eluent at a flow rate of 0.6 mL/min. Each run was 30 min at 50 °C. The presence of HEA was detected and quantified by an UV detector (DAD-3000) at wavelengths of 214 nm and 260 nm, and by a Shodex RI-101 differential refractive index detector. Chromatograms were analyzed using Chromeleon v7.1.2 (Dionex, Sunnyvale, CA, USA).

### FTIR-ATR analysis

FTIR-ATR spectra of freeze-dried lignins were recorded between 400 and 4000 cm^−1^ using a Paragon 500 Fourier transform infrared spectrometer (PerkinElmer, Waltham, MA, USA) in the attenuated total reflectance mode using 32 scans and a resolution of 4 cm^−1^.

### NMR analysis

Quantitative ^31^P NMR analyses were carried out according to a slightly modified procedure published by Granata and Argyropoulos (1995). Approximately, 40 mg of softwood kraft lignin was dissolved in an anhydrous pyridine/deuterated chloroform mixture (1.6:1 (v/v), 325 μL), and anhydrous DMF (300 μL) was added to increase the solubility of the lignin. One hundred microliters of 22 mg/mL solution of cyclohexanol in 1.6:1 (v/v) anhydrous pyridine/deuterated chloroform solvent was used as the internal standard. Fifty microliters of chromium (III) acetylacetonate solution, made of 5.6 mg in 1 mL 1.6:1 (v/v) anhydrous pyridine/deuterated chloroform, was added as the relaxation reagent. 2-Chloro-4,4,5,5-tetramethyl-1,3,2-dioxaphospholane (100 μL) was added as the phosphorylating reagent. All ^31^P NMR experiments were acquired using a Bruker Avance 600 MHz spectrometer. The ^31^P NMR data was obtained in a 5-mm tube with a pulse angle of 90° flip and pulse delay of 10 s (relaxation time) with 256 scans. The quantification limits applied were aliphatic hydroxyls (150.0–145.4 ppm), cyclohexanol (145.3–144.9 ppm), phenolic OH units (144.0–137.6 ppm), and carboxylic acids (136.0–133.6 ppm) (Meng et al. 2019).

For ^1^H NMR analysis, 20 mg of freeze-dried lignin samples were dissolved in DMSO-d_6_ in a 3-mm NMR tube and analyzed under ambient temperature using an Avance DD2 500 MHz spectrometer (Bruker Corp., Billerica, MA, USA). Spectra were recorded with 1 s relaxation delay and 128 scans. Chemical shifts are reported in parts per million (ppm).

### Lignin acetylation and gel permeation chromatography (GPC)

Prior to GPC analysis, lignin samples were acetylated according to the procedure described by Thring et al. (2006). Briefly, 1 g of oven-dried lignin was mixed at room temperature for 48 h in 40 mL of acetic anhydride/pyridine (1:1, v/v). The modified lignin was then precipitated using 50 mL of 0.1 M HCl, filtered through a 0.45-μm polytetrafluoroethylene membrane filter, and washed with 0.1 M HCl and excess amount of Milli-Q water. The residual solids (acetylated lignin) were dried at 50 °C in a vacuum oven for 24 h.

A Waters e2695 GPC system (Waters Corp., Milford, MA, USA) equipped with a RI detector was used to analyze the acetylated lignin at a flow rate of 1 mL/min, using three 300 mm × 7.8 mm Waters columns in series including 1-Styragel HR 4 THF (5–600 k Å), 2-Styragel HR 3 THF (500–30 k Å), and 3-Ultrastyragel THF (100–10 k Å). Polystyrene standards of specific molecular weights (162, 370, 580, 945, 1440, 1920, 3090, 4730, 6320, 9590, 10400, 16700, and 42400 Da) were used as external calibration standards.

### Differential scanning calorimetry (DSC)

The glass transition temperatures (*T*_g_) of the lignin sample and LHC were measured using a differential scanning calorimeter (DSC-Q100, TA Instruments, New Castle, DE, USA). Between 5 and 10 mg of freeze-dried lignin were placed on an aluminum pan. A ramp test with a heating rate of 20 °C/min under a nitrogen flow of 70 mL/min, in a heat/cool/heat cycle from − 20 to 200 °C for samples, was carried out. The second cycle was used to calculate *T*_g_.

### Acetylation and hydroxyl value of lignin using titration

The hydroxyl value of lignin before and after modification was measured according to ASTM E222-17 (ASTM 2012). 0.5 g lignin sample was dissolved in 25 mL acetic anhydride/ pyridine solution (9.5%, v/v), and it was refluxed for 90 min. The solution was then titrated with NaOH solution 0.5 meq/mL to reach pH 8.9. The same titration was performed without lignin sample, and it was considered blank. The hydroxyl number of samples was calculated according to the following equation:

$$\mathrm{Hydroxyl}\;\mathrm{number}\;(\mathrm{mg}\;\mathrm{KOH}/\mathrm g)\hspace{0.17em}=\hspace{0.17em}\frac{(A-B)\times Nt\times56.1}W$$

Where:

*A*: mL of NaOH solution required for blank

*B*: mL of NaOH solution required for sample

*Nt*: meq/mL of solution at temperature during titration

*W*: weight of sample

56.1: KOH molar mass

## Results

### Characterization of the softwood kraft lignin used in grafting reactions

**Fig. 1:**
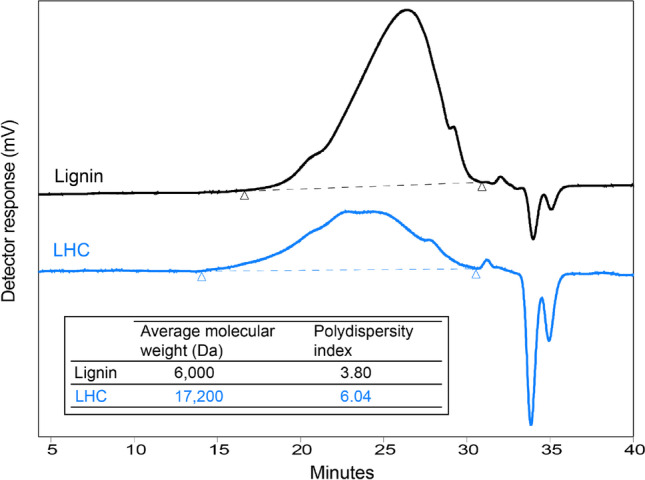
Gel permeation chromatography traces of lignin and LHC. The GPC chromatogram indicates a high polydispersity after HEA grafting compared to lignin. The average molecular weight and polydispersity of softwood kraft lignin and LHC are reported in the inserted table

^31^P NMR analysis of the softwood kraft lignin used in the study showed a lower aliphatic hydroxyl group content (1.8 mmol/g) compared to phenolic hydroxyl group content (4 mmol/g) (Table 1). As predicted, this lignin was barely soluble in water and acidic solutions (Evstigneev 2010; Glasser 2019); however, it was soluble in alkaline conditions, particularly at pH 11 or higher (Supplemental Fig. S1). The softwood kraft lignin sample had a polydispersity index of 3.8 and an average molecular weight of 6,000 Da (Fig. 1), consistent with previously reported values (Hu et al. 2016; Crestini et al. 2017; Karaaslan et al. 2021). This lignin also exhibited a low ash and mineral content (0.3%), beneficial to PU applications (Alinejad et al. 2019).

**Table 1:** Measured properties of softwood kraft lignin

Sample	Ash content (%)	Carbohydrate content (%)	Aliphatic OH (mmol/g)	Total phenolic OH (mmol/g)	COOH (mmol/g)	Total OH (mmol/g)
Softwood kraft lignin	0.3 ± 0.2^a^	4^b^	1.8	4.0	0.7	6.5

^a^Standard deviation based on five replicates

^b^Carbohydrate content calculated based on xylan and glucan contents

### HEA depletion required lignin and t-BHP and was boosted by laccase

HPLC with RI/UV detection was used to measure HEA depletion in grafting reactions with lignin. As a first step, HPLC analyses were performed to determine the optimal temperature for the grafting reaction. No significant change in HEA concentration was observed when conducting the reaction at 40 °C (Supplemental Fig. S2). When the concentration of HEA was increased 10 times to 7% (v/v) and the incubation time was extended to 72 h at 40 °C, HEA depletion up to 89% was observed (Supplemental Fig. S3). However, the concentration of HEA was reduced by 77% in the presence of lignin, t-BHP and laccase, after only 8 h at 60 °C (Supplemental Fig. S2). Furthermore, the enzyme was twice as active at 60 °C compared to 40 °C (Supplemental Fig. S4), so the grafting reaction was carried out at 60 °C. Even though the specific activity of laccase on syringol was higher at pH 8 than pH 11, the impact of pH on laccase activity was not statistically significant after pre-incubation with both HEA and t-BHP (Supplemental Fig. S5); therefore, grafting was conducted at pH 11 given the higher solubility of lignin at that pH value (Supplemental Fig. S1).

**Fig. 2:**
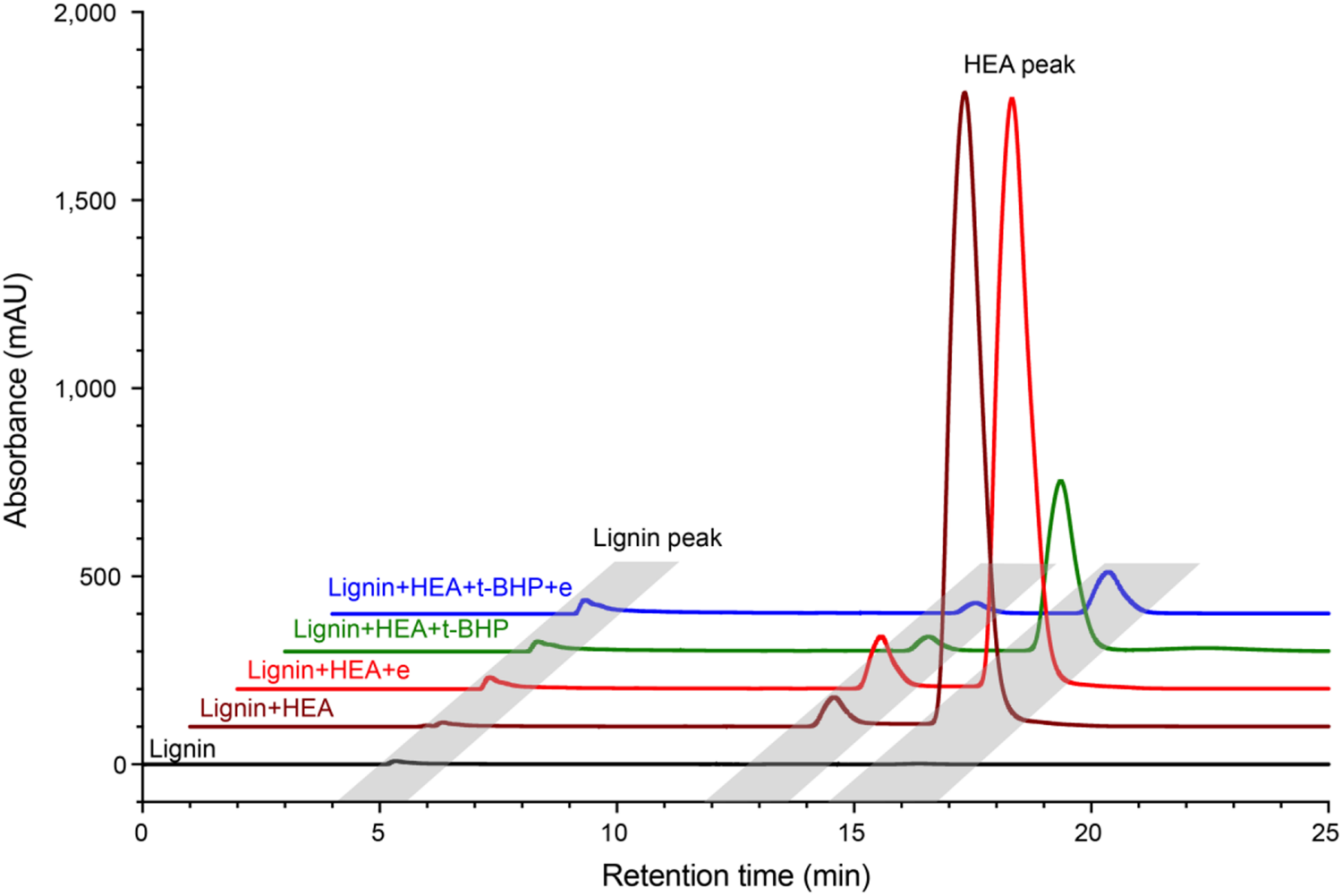
HPLC–UV analyses of HEA depletion by laccase. HEA peaks (retention times of 13.6 and 16.4 min) were detected at 214 nm; a lignin-related peak was also detected at 5.3 min. The reduction of HEA occurred in the presence of lignin and t-BHP, and it was further decreased in the addition of laccase. The 5-mL full reaction includes 7% (w/v) lignin, 0.7% (w/v) HEA, 0.7% (w/v) t-BHP, and 1U laccase (denoted as e) at pH 11, and the reaction was incubated at 60 °C for a total of 16 h

No significant degradation of HEA was observed at 60 °C and pH 11 in the presence of lignin (Supplemental Fig. S6), and HEA was stable in the presence of t-BHP (Supplemental Fig. S7). The addition of lignin to reactions containing t-BHP reduced free HEA content by up to 75% (Fig. 1). Therefore, the depletion of HEA required the addition of both lignin and t-BHP, and laccase boosted HEA consumption from 75 to 94% (Fig. 2). The depletion of HEA in the presence of lignin and t-BHP without laccase is probably due to pre-existing radicals or metal ions in the lignin substrate (Mai et al., 2001, 2002; Patil and Argyropoulos 2017).

### Increase in the average molecular weight of LHC compared to lignin

GPC analysis of the softwood kraft lignin showed that after grafting, both average molecular weight and polydispersity index increased from 6,000 to 17,200 Da and from 3.80 to 6.04, correspondingly (Fig. 1). The GPC chromatogram of LHC (Fig. 2) displays a broad dispersity profile, possibly due to grafting of HEA homopolymers with different chain lengths on lignin. To confirm the grafting of HEA to lignin, the reaction products were further analyzed by FTIR-ATR and ^1^H NMR.

### FTIR-ATR and ^1^H NMR analyses of LHC to confirm the grafting

**Fig. 3:**
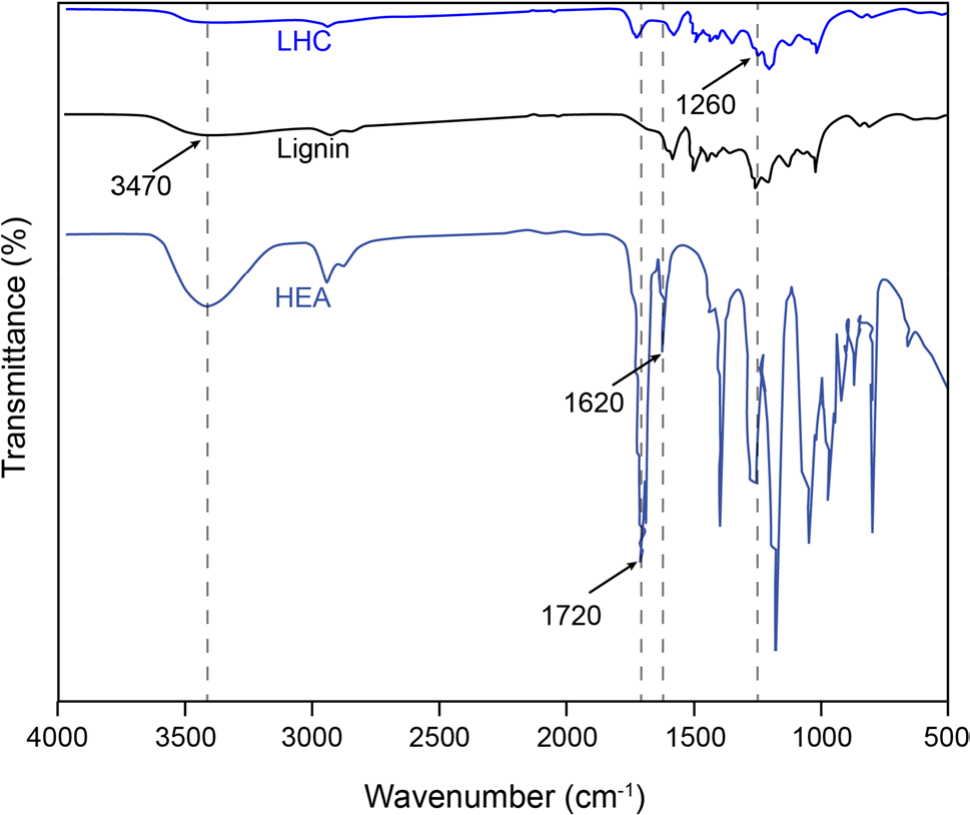
FTIR-ATR spectra of lignin, HEA monomer, and LHC product after dialysis and freeze-drying. The observed disappearance of the signal at 1,620 cm^−1^ along with emergence of a peak at 1,260 cm^−1^ indicates the grafting of HEA onto lignin (Liu et al. 2014; Sun et al. 2020, Yılmaz et al. 2017)

FTIR-ATR analyses of softwood kraft lignin before and after grafting revealed a broad absorption band at around 3,470 cm^−1^ along with two other bands at 1,590 and 1,508 cm^−1^ characteristic of hydroxyl and aromatic features of lignin (Fig. 3) (Pandey 1999). By contrast, the acrylate functionality of the HEA monomer is observed by C-O stretching at 1,250 cm^−1^ along with the -COO- absorption at 1,720 cm^−1^ (Liu et al. 2014). Accordingly, the observed disappearance of the signal at 1,620 cm^−1^ corresponding to the -C = C- absorption band (Yılmaz et al. 2017) along with emergence of a signal at 1,260 cm^−1^ (C-O) (Liu et al. 2014; Sun et al. 2020) indicates grafting between the acrylate monomer and lignin (Fig. 3).

**Fig. 4:**
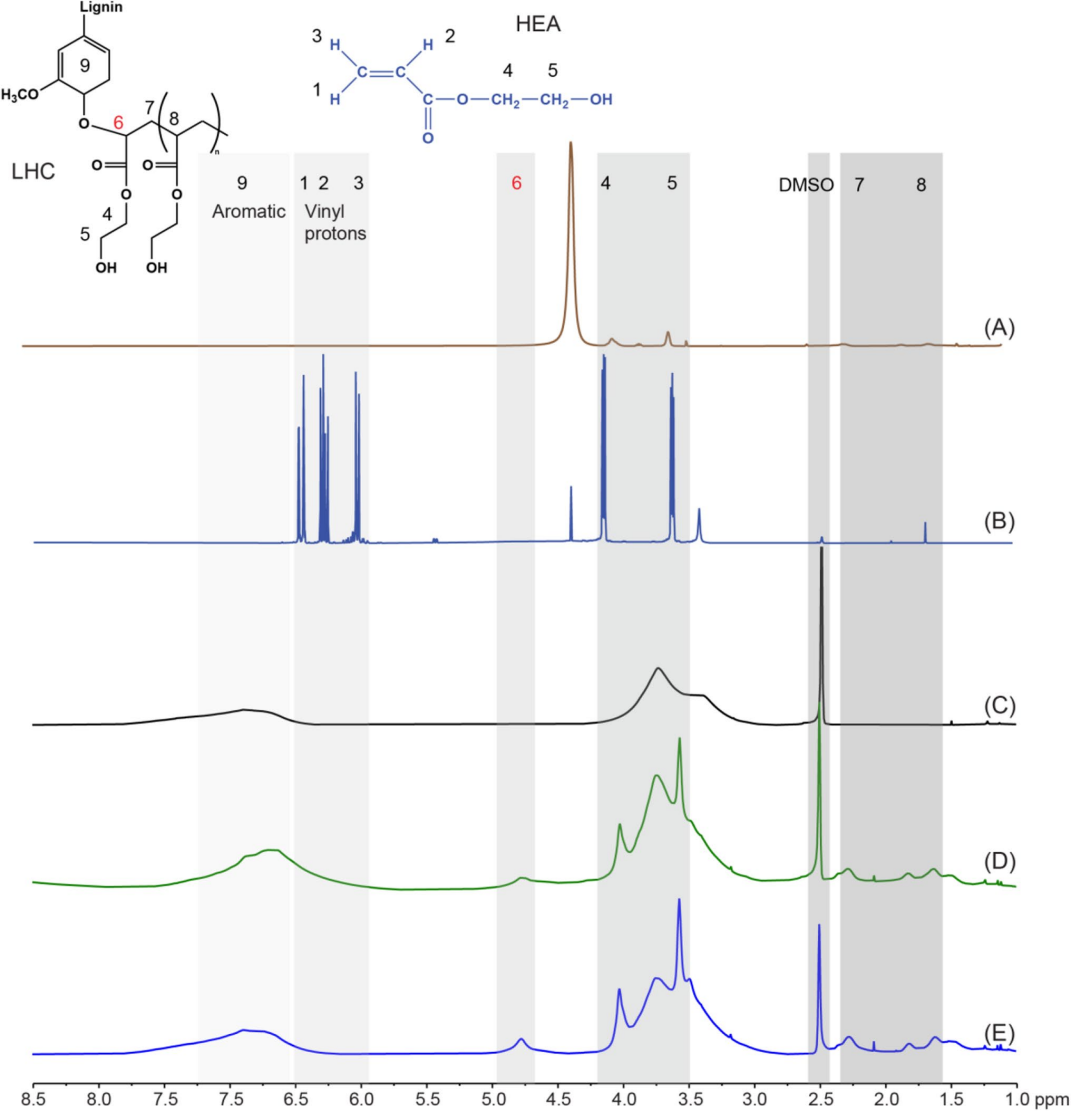
^1^H NMR analyses. **A** HEA homopolymer, **B** HEA monomer, **C** lignin, **D** lignin in the presence of initiator t-BHP and HEA, **E** LHC from the reaction with lignin, t-BHP, HEA, and laccase. Proton positions of HEA and the grafting linkage between HEA were denoted by lowercase numbers. Vinyl protons of the HEA monomer appeared at 5.5–6.5 ppm (Fig. 4B). Signals at 3.5–4.4 ppm are assigned to protons of –CH_2_ in HEA (Fig. 4B) (Vargün and Usanmaz 2005). Aromatic and methoxy proton signal peaks in lignin appeared at 6.0–8.0 ppm and 3.5–4.3 ppm (Fig. 4C), respectively (Ralph and Landucci 2010). The protons of –CH and –CH_2_ in the backbone of HEA or HEA homopolymer grafted to lignin resonate at 2.25 ppm and 1.51–1.87 ppm (Fig. 4E and F) (Vargün and Usanmaz 2005; Zong et al. 2018)

Additional structural confirmation of LHC was obtained by ^1^H NMR (Fig. 4). The chemical shift for vinyl protons (5.5–6.5 ppm) (Vargün and Usanmaz 2005; Kong et al. 2015) disappears in ^1^H NMR of LHC, confirming the occurrence of their grafting polymerization onto the lignin backbone (Fig. 4E and F). A new chemical shift at 4.7 ppm, not observed in a commercial homopolymer of HEA (Fig. 4A), further indicates an ether linkage to an aromatic ring of lignin (Liu et al. 2014; Wang et al. 2018; Zong et al. 2018). Notably, the chemical shift at 4.7 ppm and the absence of vinyl protons 5.5–6.5 ppm was also observed in reactions comprising lignin, HEA, and initiator but no laccase (Fig. 4D); however, the signal intensity at 4.7 ppm was higher in reactions comprising laccase (Fig. 4E). No characteristic chemical shifts at 4.7 ppm nor at 1.5–2.2 ppm (protons –CH and –CH_2_ in HEA backbone) were observed in the absence of lignin or t-BHP (Supplemental Fig. S8).

### Lower glass transition temperature (T_g_) of LHC compared to the untreated lignin

**Fig. 5:**
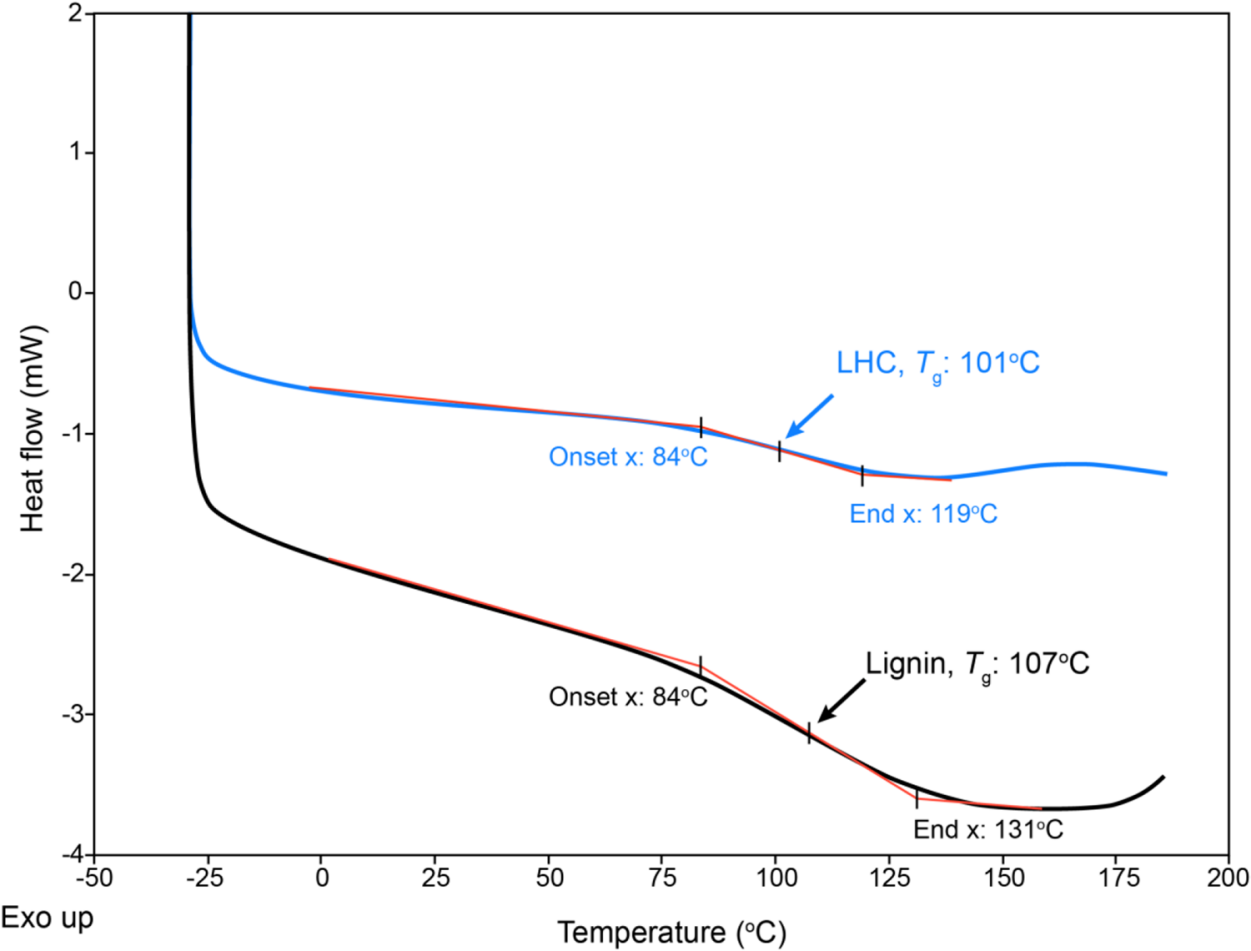
DSC analysis of lignin and LHC. Heat/cool/heat cycle from − 20 to 200 °C for samples was carried out. The second cycle was used to calculate *T*_g_. Grafting HEA to lignin reduced the *T*_g_ of the system

DSC was used to investigate the chain mobility in starting softwood lignin and LHC product. The lignin exhibited a *T*_g_ of about 107 °C, while the LHC product showed a *T*_g_ of 101 °C (Fig. 5). Notably, only one *T*_g_ was detected for the copolymer, indicating that no unreacted homopolymer or monomer remained in the LHC sample after dialysis.

### Increase in lignin hydroxyl value and reactivity toward isocyanate confirmed with titration and FTIR-ATR

**Fig. 6:**
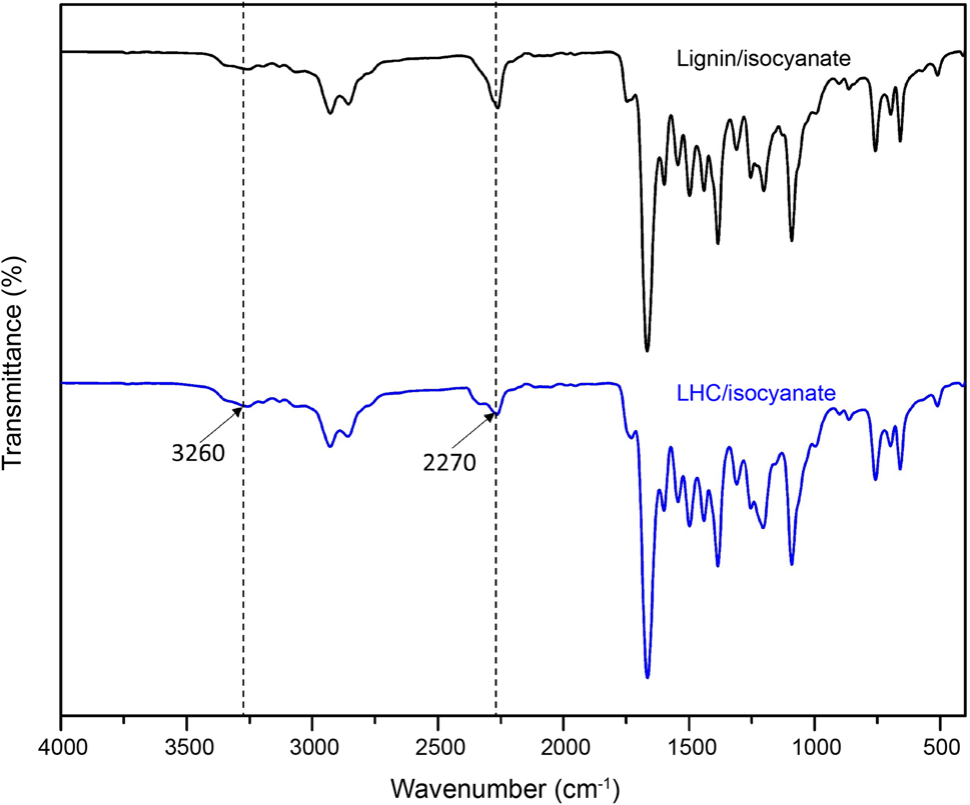
FTIR-ATR spectra of lignin and LHC samples after reaction with isocyanate. Bands at 2,270 and 3,260 cm^−1^ attribute to isocyanate and N–H in urethane, respectively

Due to low solubility of LHC in ^31^P NMR solvents, titration was used to quantify the total hydroxyl value of lignin sample before and after grafting. The titration analysis revealed that the total hydroxyl value of kraft lignin increased from 313 to 410 mg KOH/g, showing a 31% increase in the hydroxyl value of LHC. In addition, the reactivity of lignin and LHC was measured with phenyl isocyanate using FTIR-ATR. Compared to the unmodified lignin, the LHC sample showed higher reactivity toward isocyanate (Fig. 6), as indicated by a decrease in intensity of the isocyanate peak at 2,270 cm^−1^ and a slight increase in the urethane peak at 3,260 cm^−1^ (-NH stretching) (Defeyt et al. 2017).

## Discussion

The application of kraft lignin in bio-based materials is often challenged by low reactivity, structural heterogeneity, and poor solubility in most organic solvents. Moreover, aliphatic hydroxyl groups needed for PU applications often decrease during the kraft process due to condensation reactions within lignin structures (Crestini et al. 2017; Glasser 2019; Wang et al. 2019). On the other hand, the rupture of aryl–alkyl ether linkages during the kraft pulping process can increase the phenolic hydroxyl content in kraft lignin, which can promote the direct oxidation of lignin by laccases (Vuong et al. 2021). In this work, softwood kraft lignin-hydroxyethyl acrylate copolymers were successfully prepared in an alkaline aqueous condition using an alkaline-active laccase for radical formation and t-BHP as the initiator. By carrying out the grafting process at an alkaline condition where kraft lignin is more soluble, lignin loading reached 7% (w/v), which is higher than the previously reported 2–5% loadings of lignosulfonate (Mai et al. 2000a), acetic acid lignin and biobutanol lignin (Zong et al. 2018), and alkali lignin (Sun et al. 2020). The FTIR-ATR results are in agreement with the previously reported application of FTIR-ATR to demonstrate laccase-mediated grafting of acrylic acid to lignosulfonate at pH 4.5, where successful grafting was shown by the appearance of a peak at around 1,720 cm^−1^, corresponding to unconjugated carbonyls (Yu et al. 2017). Furthermore, the disappearance of ^1^H NMR peaks corresponding to vinyl protons (5.5–6.5 ppm) and the appearance of a new peak (4.7 ppm) in the LHC product indicated the successful HEA graft polymerization onto the lignin backbone.

Reactions comprising HEA, t-BHP, and laccase alone confirmed that HEA polymerization did not occur without lignin. This finding is in agreement with the role of lignin in providing phenoxy radicals produced through enzymatic oxidation by laccase (Mai et al. 2002). HEA graft polymerization onto lignin in the absence of laccase, albeit to a lesser extent, likely resulted from the presence of organic radicals and metal ions in the lignin substrate that decompose t-BHP. For example, residual iron (Fe^2+^) in ash was reported to decompose t-BHP into radicals (Mai et al. 2001). t-BHP decomposition can be also initiated by stable organic radicals in lignin such as semiquinone radicals or semiquinone radical anions that are generated as the results of biological effects (fungal attack), chemical processes (alkaline extraction), mechanical process (milling), and photochemical reactions (photo-oxidation) (Patil and Argyropoulos 2017).

Production of LHC required the addition of the t-BHP initiator. This result is consistent with the earlier observation that phenoxy radicals did not initiate the polymerization of acrylic monomers (Mai et al. 2000b) and reinforces the proposed mechanism shown in Scheme 1, where laccase produces phenoxy radicals on lignin, which leads to the formation of alkoxy or peroxy radicals from t-BHP, initiating HEA polymerization and consequently lignin grafting.

The increase in average molecular weight after grafting indicates a higher average molecular weight and wider molecular weight distribution due to the grafted HEA polymer chains onto lignin. The partial polymerization of lignin through catalytic oxidation by laccase and coupling of phenoxy radicals could also contribute to the increase in molecular weight (Agustin et al. 2021; Wang et al. 2021). The lower *T*_g_ as observed for LHC compared to kraft lignin was expected, as grafted homopolymers onto lignin would facilitate the movement of the copolymer compared to lignin (Sun et al. 2020). Additionally, grafting aliphatic hydroxyl groups could further reduce the *T*_g_ of the system (Cui et al. 2013). However, the slightly lower *T*_g_ value of LHC compared to kraft lignin points to the possible grafting of short-chain homopolymers and HEA monomers to lignin rather than longer chain homopolymers. The *T*_g_ of LHC (101 °C) is much higher than that of HEA homopolymer, which was reported to be − 25 to − 9 °C (Vargün and Usanmaz 2005), indicating the incorporation of the relatively rigid lignin. In summary, ^1^H NMR and FTIR-ATR confirmed the successful grafting of HEA onto lignin, which increased the total hydroxyl value of the grafted lignin copolymer and its reactivity toward isocyanate. This demonstration opens new possibilities for alkaline-active laccases and potentially other lignin-active enzymes in the manufacturing of bio-based polyurethanes.

## Supplementary information

Below is the link to the electronic supplementary material.

Supplementary file1 (PDF 3636 KB)

## Data Availability

All data generated or analyzed during this study are included in this published article [and its supplementary information files].
