# Supplementary material for: Enzymatic synthesis of kraft lignin-acrylate copolymers using an alkaline tolerant laccase
Source: Appl Microbiol Biotechnol. 2022 Apr 22;106(8):2969–79. doi: 10.1007/s00253-022-11916-z (PMC9064866; doi:10.1007/s00253-022-11916-z)
Supplement: Supplementary file 1 — Supplementary file1 (PDF 3636 KB) [file 253_2022_11916_MOESM1_ESM.pdf]

Supplementary Information (SI)

Enzymatic synthesis of kraft lignin-acrylate copolymers using an alkaline tolerant laccase

Maryam Arefmanesh[1a], Thu V. Vuong [1a], Saeid Nikafshar[2], Henrik Wallmo[3], Mojgan Nejad[2,4], and Emma R. Master[1,5*]

[1]Department of Chemical Engineering and Applied Chemistry, University of Toronto, 200 College Street, Toronto, Ontario, M5S 3E5, Canada

[2]Department of Forestry, Michigan State University, 480 Wilson Road, Michigan, 48824, United States

[3]Valmet AB, Regnbågsgatan 6, PO Box 8734, SE-402 75 Gothenburg, Sweden

[4]Department of Chemical Engineering and Material Science, Michigan State University, 428 S Shaw Lane, Michigan, 48824, United States

[5]Department of Bioproducts and Biosystems, Aalto University, FI-00076 Aalto, Kemistintie 1, Espoo, Finland

[a]These authors have contributed equally to this work and share first authorship

*Corresponding author:

Emma R. Master

Telephone: 416-946-7891

Email: emma.master@utoronto.ca

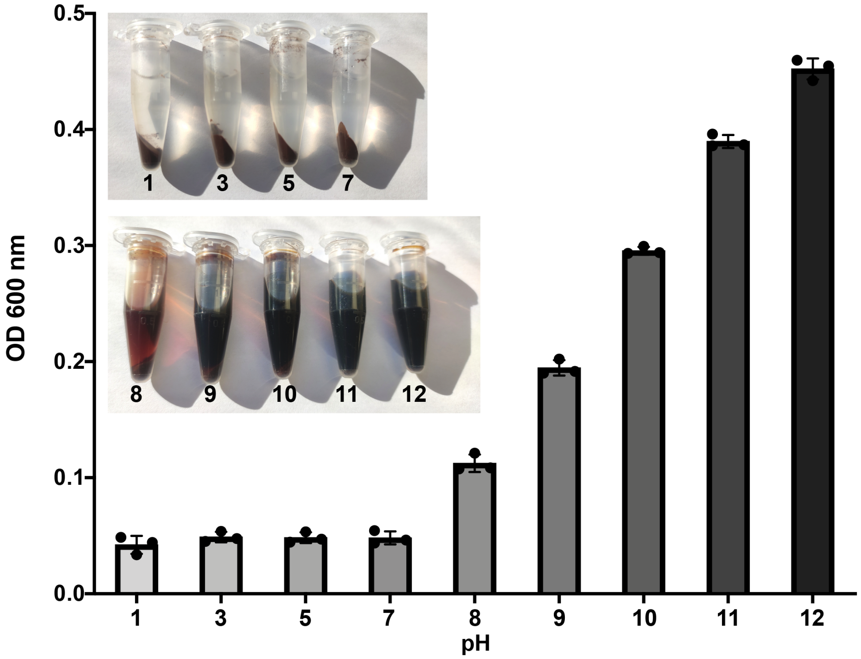

Fig. S1 Solubility of softwood kraft lignin. Dried lignin was suspended in pH 1 to pH 12. The samples were centrifuged at 16,000 x g for 5 min (inserted images) and then the absorbance of diluted supernatant was measured at 600 nm.

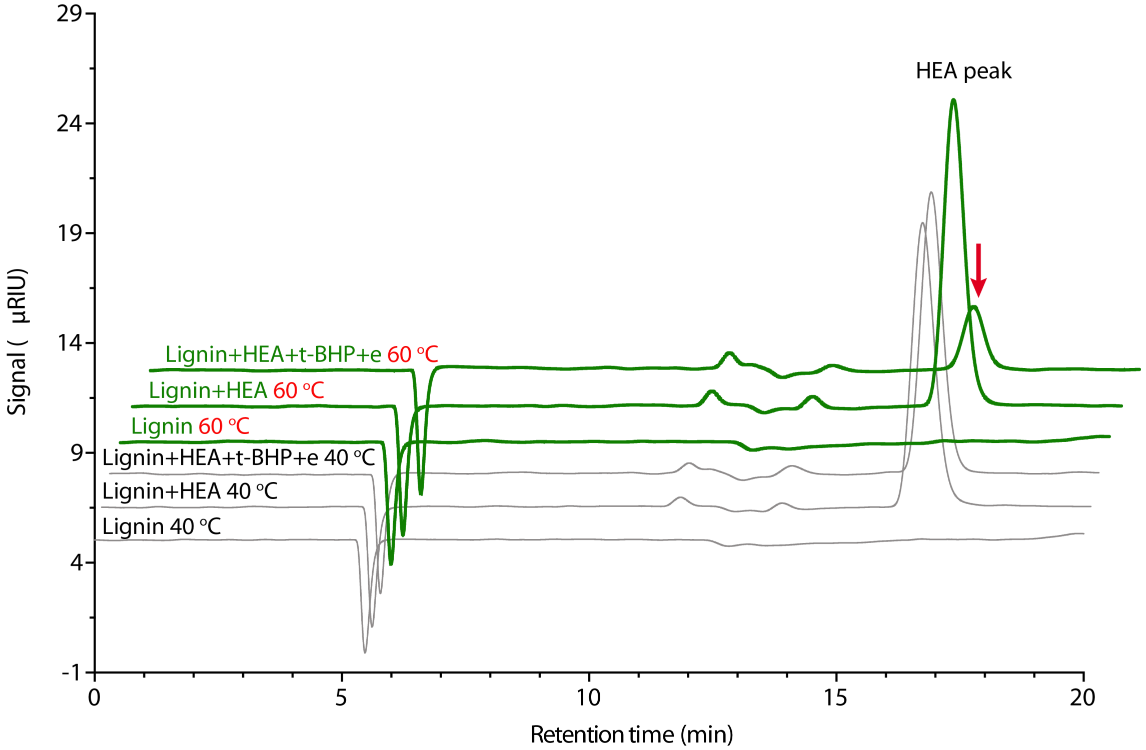

Fig. S2 Impact of temperature on HEA depletion. Compared to reactions performed at 40 °C (grey lines), reactions performed at 60 °C (green lines) significantly reduced the HEA signal, as measured by HPLC-RI, after 8-h incubation. The total reaction volume was 5 mL and comprised 7 % (w/v) lignin, 0.7 % (w/v) HEA, 0.7 % (w/v) t-BHP and 1U laccase (denoted as e) at pH 11. HEA depletion is highlighted by the red arrow.

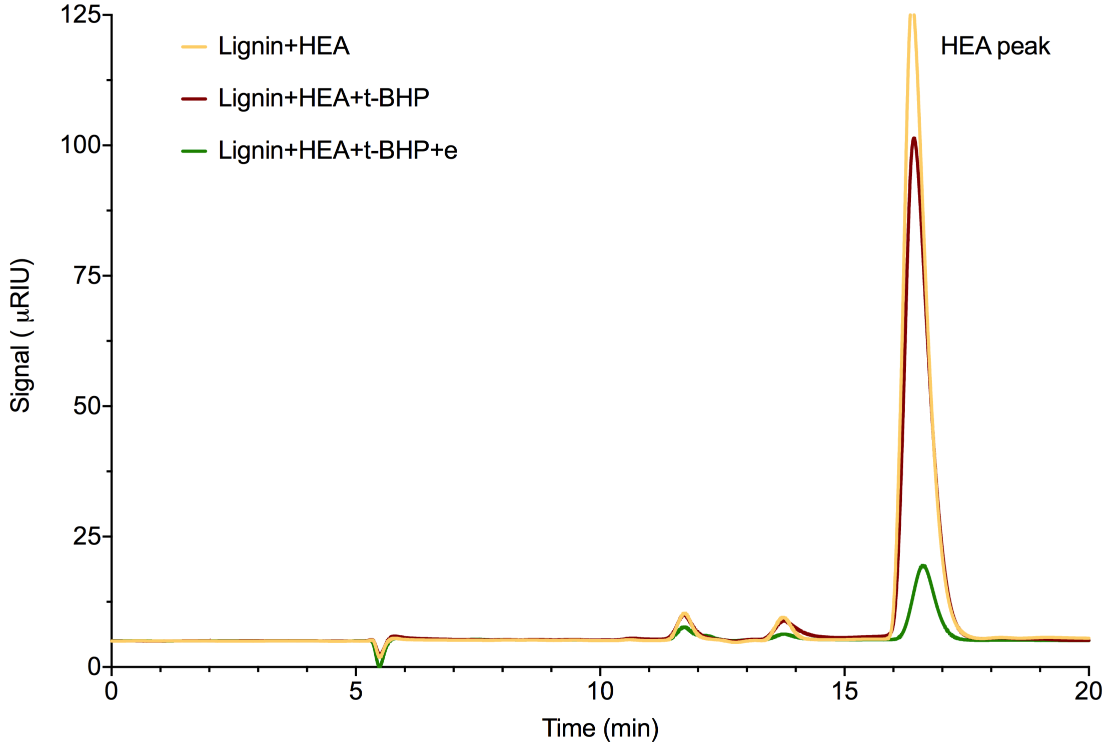

Fig. S3 Effect of 72 h-incubation time at 40 °C on HEA depletion. The depletion of HEA, as measured by HPLC-RI, in 5-mL reactions comprising 7 % (w/v) lignin, 7 % (w/v) HEA, 0.7 % (w/v) t-BHP and 1U laccase (denoted as "e") at pH 11 after 3 days at 40 °C.

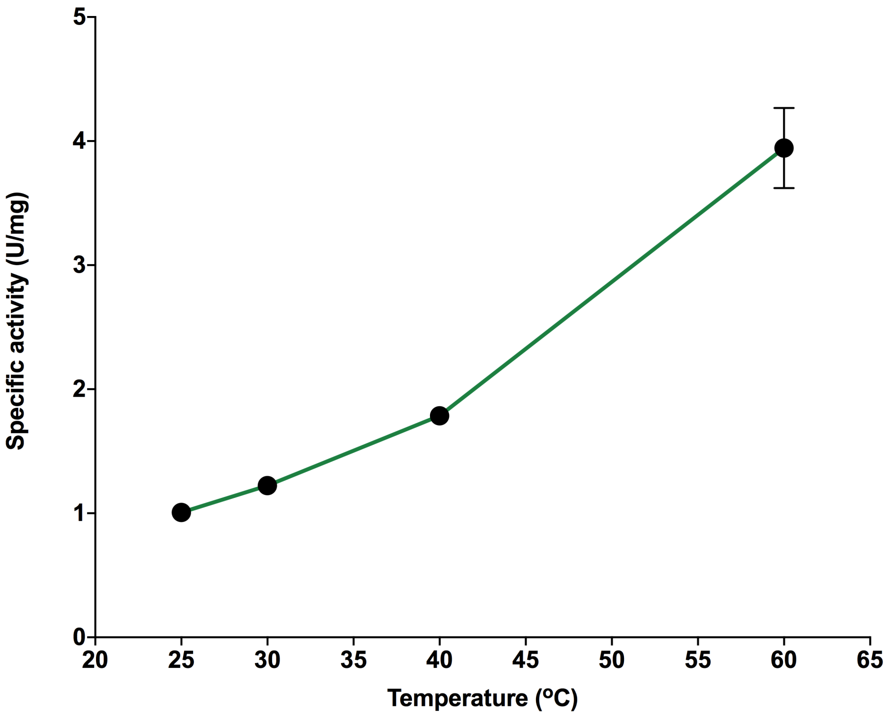

Fig. S4 Temperature profile of laccase. Activity of 5 μg/mL laccase on 10 mM 2,6-dimethoxyphenol (DMP) was measured at 25, 30, 40 and 60 °C, and expressed as U/mg of protein, where U is μmol of product coerulignone /min.

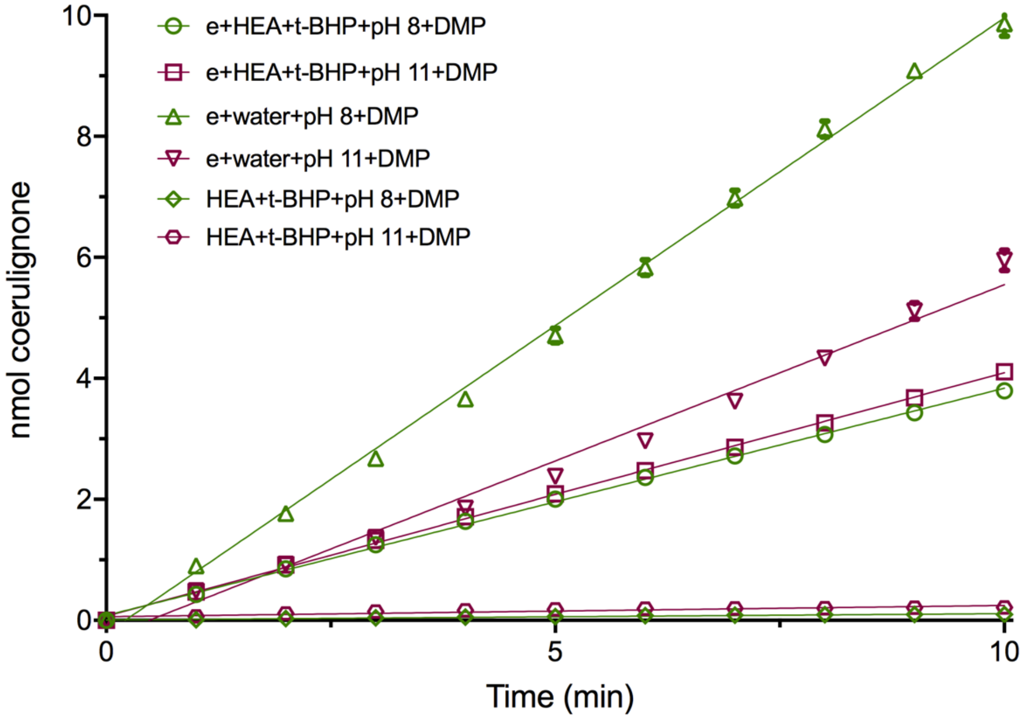

Fig. S5 Laccase activity after pre-incubation with HEA and t-BHP at 60 °C and pH 11. Laccase was pre-incubated with 0.7 % (w/v) HEA and 0.7 % (w/v) t-BHP at pH 8 and pH 11 (50 mM Tris-buffer) in 1-mL reactions at 60 °C for 4 h, before being assayed on 10 mM DMP, the final concentration of laccase was 5 μg/mL.

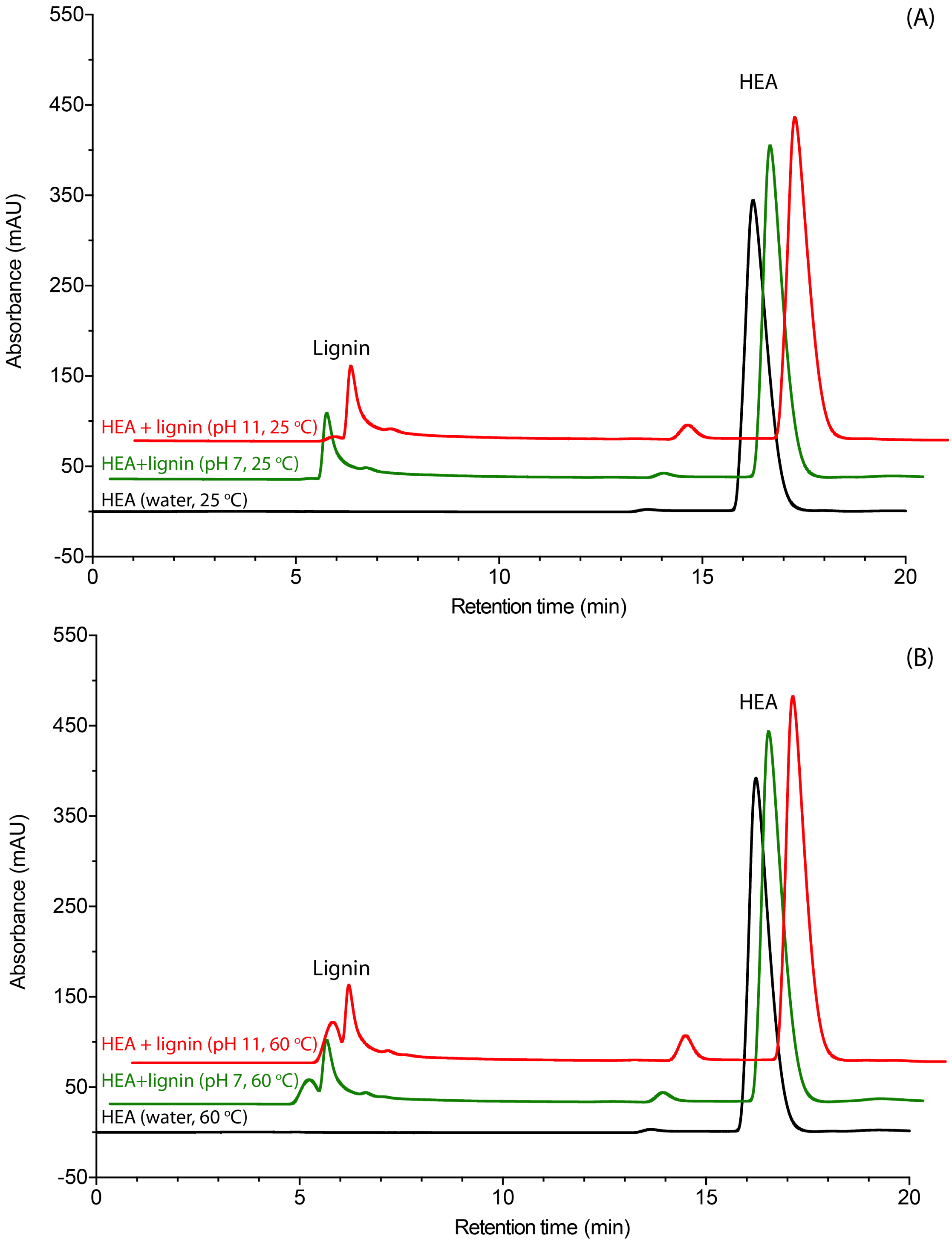

Fig. S6 Effect of pH and temperature on HEA degradation. HEA alone (0.7 %, w/v, the main peak at the retention time of 16.4 min) or in the presence of lignin (7 %, w/v) at pH 7 or pH 11 in 5-mL reactions were incubated at 60 °C for 16 h; the presence of HEA was analyzed by HPLC-UV with the wavelength of 260 nm.

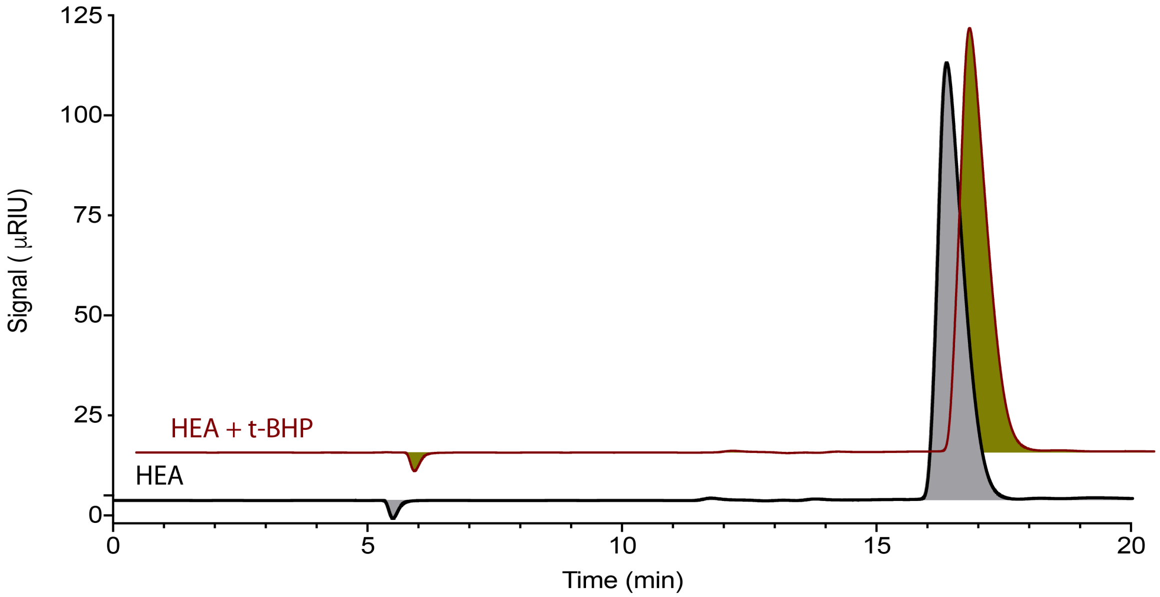

Fig. S7 No HEA depletion in the absence of lignin and laccase. HEA alone (7 %, w/v), or HEA and t-BHP (0.7 %, w/v) in 5-mL reactions was incubated at 60 ^{o}C for 16 h, and the presence of HEA was analyzed by HPLC-RI.

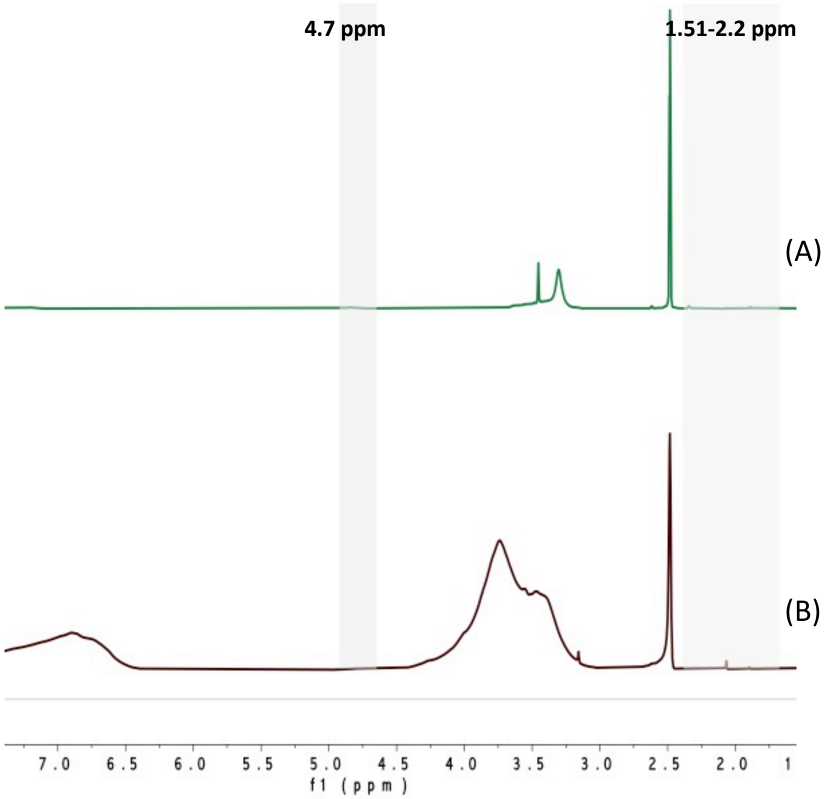

Fig. S8 ^{1}H NMR of grafting reaction. No characteristic chemical shifts (1.5-2.2 ppm and 4.7 ppm) were seen in the highlighted sections of A) laccase + HEA + t-BHP (i.e. in the absence of lignin), and B) lignin + HEA + laccase (i.e. in the absence of the initiator t-BHP).
